# How Tilting the Head Interferes With Eye-Hand Coordination: The Role of Gravity in Visuo-Proprioceptive, Cross-Modal Sensory Transformations

**DOI:** 10.3389/fnint.2022.788905

**Published:** 2022-03-10

**Authors:** Jules Bernard-Espina, Daniele Dal Canto, Mathieu Beraneck, Joseph McIntyre, Michele Tagliabue

**Affiliations:** ^1^Université de Paris, CNRS, Integrative Neuroscience and Cognition Center, Paris, France; ^2^Ikerbasque Science Foundation, Bilbao, Spain; ^3^TECNALIA, Basque Research and Technology Alliance (BRTA), San Sebastian, Spain

**Keywords:** multisensory integration, cross-modal transformation, gravity, reaching/grasping movement, eye-hand coordination, vision, proprioception, otolith

## Abstract

To correctly position the hand with respect to the spatial location and orientation of an object to be reached/grasped, visual information about the target and proprioceptive information from the hand must be compared. Since visual and proprioceptive sensory modalities are inherently encoded in a retinal and musculo-skeletal reference frame, respectively, this comparison requires cross-modal sensory transformations. Previous studies have shown that lateral tilts of the head interfere with the visuo-proprioceptive transformations. It is unclear, however, whether this phenomenon is related to the neck flexion or to the head-gravity misalignment. To answer to this question, we performed three virtual reality experiments in which we compared a grasping-like movement with lateral neck flexions executed in an upright seated position and while lying supine. In the main experiment, the task requires cross-modal transformations, because the target information is visually acquired, and the hand is sensed through proprioception only. In the other two control experiments, the task is unimodal, because both target and hand are sensed through one, and the same, sensory channel (vision and proprioception, respectively), and, hence, cross-modal processing is unnecessary. The results show that lateral neck flexions have considerably different effects in the seated and supine posture, but only for the cross-modal task. More precisely, the subjects’ response variability and the importance associated to the visual encoding of the information significantly increased when supine. We show that these findings are consistent with the idea that head-gravity misalignment interferes with the visuo-proprioceptive cross-modal processing. Indeed, the principle of statistical optimality in multisensory integration predicts the observed results if the noise associated to the visuo-proprioceptive transformations is assumed to be affected by gravitational signals, and not by neck proprioceptive signals *per se*. This finding is also consistent with the observation of otolithic projections in the posterior parietal cortex, which is involved in the visuo-proprioceptive processing. Altogether these findings represent a clear evidence of the theorized central role of gravity in spatial perception. More precisely, otolithic signals would contribute to reciprocally align the reference frames in which the available sensory information can be encoded.

## Introduction

When reaching to grasp an object, arm proprioceptive signals and the visually acquired object position/orientation must be compared. A typical situation in which visuo-proprioceptive communication is strictly necessary is at the beginning of the reaching movement if the hand is out of sight. There are, however other common situations where cross-modal transformations, i.e., the encoding of visual information in a proprioceptive space and vice-versa, is necessary during the whole reaching movement: for instance, when trying to insert a bolt from beneath a plate on which the threaded hole location is visually identified from above. There is also evidence that the visuo-proprioceptive interaction is performed even when it is not strictly necessary, that is even when object and hand can be both seen, or both sensed through proprioception, before the movement onset (Sober and Sabes, 2005; Sarlegna and Sainburg, 2007, 2009; Sarlegna et al., 2009) and during movement execution (Tagliabue and McIntyre, 2011, 2013, 2014; Cluff et al., 2015; Crevecoeur et al., 2016; Arnoux et al., 2017).

It has been shown that tilting laterally the head when seating interferes with the communication between visual and proprioceptive systems (Burns and Blohm, 2010; Tagliabue and McIntyre, 2011) and we demonstrated that this phenomenon is independent from the phase of the movement during which the head is tilted (Tagliabue et al., 2013; Tagliabue and McIntyre, 2014). These studies, however, did not allow understanding whether the neck on trunk lateral flexion *per se* (the signals originating from the neck muscles), or the head misalignment with respect to the vertical (gravitational signals), interferes with cross-modal transformations. The first option, that we call here the *Neck Hypothesis*, would be consistent with the contribution of the neck flexion angle information to the kinematic chain linking the hand to the eyes and that may be thus used to compute visuo-proprioceptive transformations (Sabes, 2011). This hypothesis has two possible variants: “Neck1 Hp,” wherein the lateral neck flexions *per se* interferes with eye-hand transformation, because of the rarity of adopting such neck postures when performing reaching/grasping tasks; “Neck2 Hp,” wherein lateral neck flexions require an increase of the muscle activations to support the weight of the head, resulting in increased signal-dependent noise that would interfere with eye-hand transformations (Abedi Khoozani and Blohm, 2018). An alternative option, called here the *Gravity Hypothesis* (Gravity Hp), is related to the idea that gravity might play a fundamental role in the reciprocal calibration between visual and proprioceptive senses (Paillard, 1991), since it can be both seen (the visual environment provides information about the vertical) and felt (mechano-receptors detect gravity action). The head-vertical misalignment might hence perturb the ability of using gravity as reference for visuo-proprioceptive transformations. This could be due to an increase of the otolithic noise with the lateral head tilt (Vrijer et al., 2008) or to the fact that eye-hand coordination tasks are more commonly performed with the head straight and sensorimotor precision has been shown to be proportional to the task usualness (Howard et al., 2009).

To discriminate between these hypotheses, we performed a first virtual reality experiment in which the subject had to perform in a *Seated* and in a *Supine* position the same cross-modal task: align the hand to “grasp” a visual target with the unseen hand (Tagliabue and McIntyre, 2011; Tagliabue et al., 2013). To test the effect of the neck flexion, the subjects are asked to laterally tilt the head between the target acquisition and the hand movement onset. If “*Neck1 Hp*” is correct, the subjects’ performance should not change notably between postures, because the tasks performed in the seated and supine condition do not significantly differ in terms of lateral neck flexion. On the other hand, “*Neck2 Hp*” predicts an improvement of the precision when supine, because, thanks to a special head support, in this position the neck muscles never have to sustain the head weight, resulting in spindle-noise reduction (Abedi Khoozani and Blohm, 2018). Neck proprioceptive degradation is not to be expected with the head-support, because there is evidence that a decrease of the muscle tone, as experienced by astronauts in weightlessness, does not reduce the sensitivity of the muscle receptors (Roll et al., 1993). Finally, “*Gravity Hp*” will be supported by a decrease of precision when supine, because when lying on their back the subject’s head is misaligned with respect to gravity during the whole task and not only during the response phase, as in the seated configuration.

Two control experiments were performed to test whether potential effect of posture observed in the cross-modal task could be due to an effect of posture on visual and/or proprioceptive perception, and not on the sensory transformations. In the first control experiment the subjects performed a unimodal visual task: only vision could be used for both target acquisition and response control. In the second control experiment a unimodal proprioceptive task was tested: both target and response could be sensed through proprioception only.

In order to compare the *Neck* and *Gravity Hypotheses* predictions with the measured subjects’ precision and sensory weighting, we applied our “*Concurrent Model*” (see below) of multisensory integration (Tagliabue and McIntyre, 2008, 2011, 2012, 2013, 2014; Tagliabue et al., 2013; Arnoux et al., 2017; Bernard-Espina et al., 2021) to the cross- and uni-modal tasks tested here.

To confirm our interpretation of the first set of results, we performed an additional experiment in which the subjects were tested seated and supine, but without lateral neck flexions. The goal was to specifically test the effect of the modulation of the gravitational information without interference from neck muscle-spindles’ signals.

## Materials and Methods

### Ethics Statement

The experimental protocol was approved by the Ethical Committee of the University of Paris (N° CER 2014-34/2018-115) and all participant gave written informed consent in line with the Declaration of Helsinki.

### Experimental Setup and Procedure

The setup is very similar to what used in our previous studies (Tagliabue and McIntyre, 2011, 2012), consisting of the following components: an active-marker motion-analysis system (CODAmotion; Charnwood Dynamics) used for real-time recording of the three-dimensional position of 19 infrared LEDs (sub-millimeter accuracy, 200-Hz sampling frequency). Eight markers were distributed ∼10 cm apart on the surface of stereo virtual reality goggles (nVisor sx60, NVIS) worn by the subjects (field of view: 60°, frame rate: 60 Hz, resolution: 1,280 × 1,024 pixels, adjustable inter-pupillary distance); eight on the surface of a tool (350 g, isotropic inertial moment around the roll axis) that was attached to the subjects’ dominant hand; and three attached to a fixed reference frame placed in the laboratory. Custom C++ code was developed by the research team to optimally combine the information about the three-dimensional position of the infrared markers and the angular information from an inertial sensor (IS-300 Plus system from InterSense) placed on the VR headset to estimate in real-time the position and the orientation of the subject’s viewpoint and thus to update accordingly the stereoscopic images shown in the virtual reality goggles. For tracking the hand movement only infrared markers were used.

**FIGURE 1:**
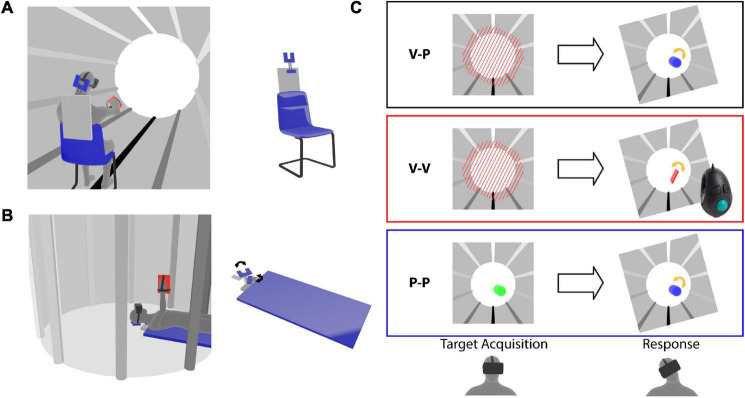
Virtual reality experimental paradigm. Representation of the **(A)** Seated and **(B)** Supine conditions. The subjects wear a virtual reality headset and a tool is fixed to their hand. The left images illustrate the virtual tunnel in which the subject perform the task. The configuration of the rotating head support (forked structure) is shown for the two postural conditions. **(C)** Target presentation (left) and response modality (right) for the three experiments. The tilted frames in the response phase represent the lateral neck flexion that the subjects perform after the target memorization. For the cross-modal (V-P) task the target is represented by tilted red bars and during the response the subject hand movements are applied to a blue capsule, which provides visual feedback about the pointing direction in pitch and yaw, but no visual cues about the prono-supination of the hand used to reproduce the target orientation. For the unimodal visual (V-V) task the target is presented as in the V-P task, but a virtual hand-tool (red rectangle) controlled by a trackball is used to reproduce the target orientation. During the target acquisition of the unimodal proprioceptive (P-P) task the color of the capsule representing the subject hand changes from red to green when the hand approaches the target orientation. The response modality is the same as in the V-P task.

The three-dimensional virtual environment shown to the subjects through the head mounted display consisted of a cylindrical tunnel (Figure 1). Longitudinal marks parallel to the tunnel axis were added on the walls to help the subjects to perceive their own spatial orientation in the virtual word. The fact that the marks went from white in the “ceiling” to black on the “floor” facilitated the identification of the visual vertical.

### Experimental Paradigm

The task consisted of three phases: (1) memorization of the target orientation, 2) lateral neck flexion, and (3) alignment of the tool to the remembered target orientation. As in our previous studies (Tagliabue and McIntyre, 2011, 2012, 2013, 2014; Tagliabue et al., 2013; Arnoux et al., 2017), we took advantage of the head rotation to introduce a sensory conflict with the subjects not noticing it (see below). The target could be laterally tilted with respect to the virtual vertical of −45°, −30°, −15°, 0°, +15°, +30° or +45°. The subjects had 2.5 s to memorize its orientation. After the target disappeared, the subject was guided to laterally tilt the head 15° to the right or to the left by a sound with a left-right balance and a volume corresponding to the direction and the distance from the desired inclination. If the subject was unable to extinguish the sound within 5 sec, the trial was interrupted and repeated later on, otherwise a go signal was given to indicate that he/she had to reproduce the target orientation with the tool. The subject clicked on the trigger of a trackball held in the hand to validate the response.

In order to quantify the sensory weighting in each experimental condition a sensory conflict was artificially introduced (Tagliabue and McIntyre, 2011): tracking the virtual reality goggles was normally used to hold the visual scene stable with respect to the real world during the lateral head tilt, but in half of the trials, a gradual, imperceptible conflict was generated such that, when the subjects laterally flex the neck, they received visual information corresponding to a larger head tilt. The amplitude of the angle between the visual vertical and subject body axis varied proportionally (by a factor of 0.6) with the actual head tilt, so that for a 15° lateral head roll a 9° conflict was generated. When, at the end of the experiment, the subjects were interviewed about the conflict perception, none of them reported to have noticed the tilt of the visual scene.

Each subject was tested in two postural conditions: Seated and Supine (Figures 1A,B). In order to compensate for possible learning effects, half of the subjects were tested first seated and then supine, and the other half in the opposite order. When the subjects performed the task in the supine position, they lay in a medical bed with their head supported by an articulated mechanical structure allowing for lateral neck flexions (Figure 1B). When the subject performed the task in a seated position the same head support was fixed to the back of the chair to restrain the head movements in a way similar to the supine condition (Figure 1A). Since the main axis of the virtual tunnel always corresponded to the anterior-posterior subject direction, it was horizontal and vertical in the Seated and Supine Condition, respectively.

As detailed below, the first three experiments presented in this study differed only by the sensory information available to acquire the target and to control the tool during the response (Figure 1C). The task used in the fourth, additional experiment was the same as for the main cross-modal experiment with the exception that the subject always kept the head aligned to the body.

#### Cross-Modal Experiment

The target was presented visually and during the response the tool orientation could be controlled through arm proprioception only (V-P task). As shown in the top part of Figure 1C, the target consisted of parallel beams blocking the tunnel in front of the subject. In the response phase, subjects raised their hand and reproduced the memorized beams orientation by prono-supinating the palm. The subjects’ hands were represented in the virtual environment as a capsule with the same main axis so that all its degrees of freedom except the roll (hand prono-supination) could be visually controlled. It follows that only arm proprioception could be used to control the alignment task.

#### Uni-Modal Visual Experiment

Both target acquisition and tool control orientation could be performed by using vision only (V-V task). The target was represented by the beams as in Experiment 1. For the response, subjects did not move the hand, which was kept next to the body. A virtual representation of the tool fixed to the subject hand appeared in front of their eyes with a random roll orientation (see middle part of Figure 1C). They used a trackball to change its roll angle and to align it to the memorized beams. In this way only visual information could be used to evaluate the task achievement.

#### Uni-Modal Proprioceptive Experiment

Both target and tool orientation could be sensed through proprioception only (P-P task). The beams were not shown to the subjects. To sense the target orientation, they raised the hand, which was represented by a capsule, as in the response phase of Experiment 1. In this phase the color of the capsule changed as a function of the hand roll turning from red to green as the hand approached the target roll angle. Thus, subjects had to pronate or supinate the hand to find the target orientation. After 2.5 s with the correct hand orientation an audio signal instructed the subject to lower the arm. The only information available to memorize the roll orientation of the target was the proprioceptive feedback related to forearm pronation–supination. The target orientation was in this way presented proprioceptively, without any visual feedback about the desired orientation. The response was controlled using proprioception only, as in Experiment 1.

In total 54 subjects were tested, 18 for each experiment (average age: V-P 26.5 ± 9; V-V 30 ± 6; P-P 24.5 ± 6). The number of male and female participants was balanced and about 17% of the subjects were left-handed. The subjects performed two trials for each combination of target orientation, head inclination and sensory conflict, for a total of 56 (= 2 × 7 × 2 × 2) trials per posture. The order of the trials was randomized.

#### Neck Straight Experiment

The task is very similar to the one tested in the “Cross-modal Experiment” except that the subjects were not asked to laterally flex the neck after the target memorization. Twelve subjects participated to the experiment (age: 38.5 ± 8). Half of them performed the Seated condition before the Supine condition, the other half did the opposite to compensate for possible learning effects. As for the previous experiment, each target orientation was tested twice per postural condition, for a total of 28 (= 2 × 2 × 7) responses. The head mounted display used for these tests was an Oculus Rift (field of view: 90°, frame rate: 90 Hz, resolution: 1,080 × 1,200 pixels, adjustable inter-pupillary distance). As for the main experiments, a custom C++ code was developed by the research team to integrate optical (Codamotion system) and inertial (embedded in the Oculus-Rift) sensors and to update the stereo images provided in virtual reality headset.

### Data Analysis

The subjects’ performance was analyzed using Matlab (MathWorks, RRID: SCR_001622) in terms of the lateral inclination (roll) of the tool when they validated the response. In order to describe the variability of the subject responses, we computed the root mean square of the difference, *RMSd*, between the two responses, *r*, to each combination of target, *t*, and head, *h*, inclination in the trials without conflict.


(1) $$R\,M\,S\,d=\sqrt{\frac{\sum_{h=1}^{2}\sum_{t=1}^{7}{\left(r_{t,h,1}-r_{t,h,2}\right)}^{2}}{14}}$$

**FIGURE 2:**
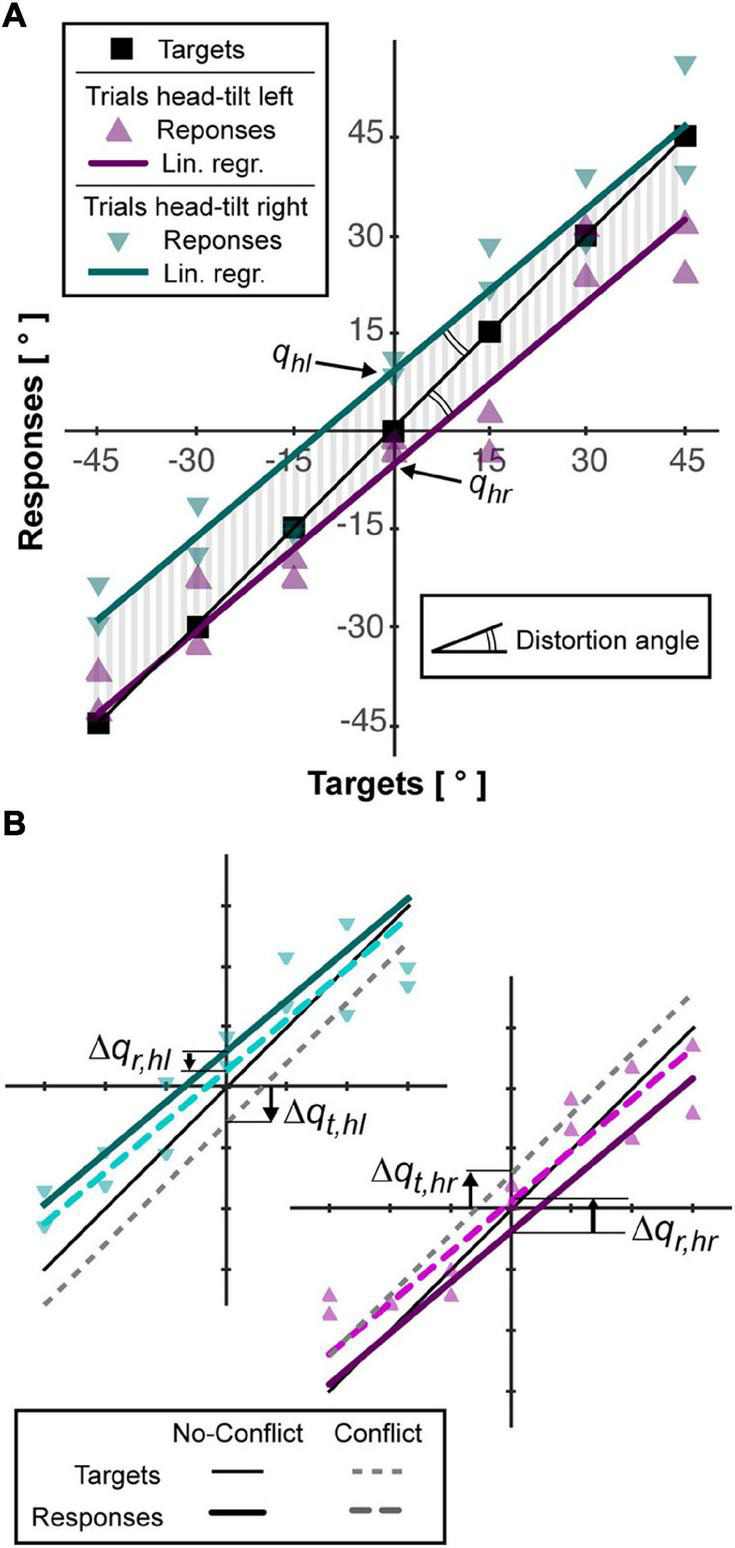
Example of subject responses and associated analysis. **(A)** The responses for trials without conflict (triangles) are linearly interpolated as describe in “Materials and Methods” section (colored lines). The area (vertical gray bars) between each interpolation line and the line joining the targets (squares) is used to compute accuracy, *Acc*. The angle between the interpolation lines and the targets line is used quantify the distortion, *Dist*, of the responses. The intersections between the vertical axis and the lined interpolating the responses after left and right neck flexion (*q*_*hl*_, *q*_*hr*_) are used to quantify the response bias induced by the head roll (Aubert-Müller effect, *AMe*). **(B)** For the trials with conflict, that is, rotation of the visual scene, the responses after left and right flexion of the neck are interpolated separately (see “Materials and Methods” section) and represented by dotted, colored lines. To estimate the relative importance given to the visual information, the vertical distance between the lines interpolating the response with and without conflict, *Δq_*r,hl*_* and *Δq_*r,hr*_*, is computed and compared to the theoretical deviation of the targets, if they assumed to move together with the visual scene, *Δq_*t,hl*_* and *Δq_*t,hr*_*.

To describe the characteristics of the average behavior of the subjects, the linear regression lines of their responses after tilting the head to the right and to the left were computed imposing their parallelism (see Figure 2A). Each of the two regression lines have the form *r = mt+q_*i*_*, where *r* and *t* are the response and target orientation, respectively. The parameter “*m*,” common for the two lines, represents their slope. The intersection with the response axis “*q*_*i*_” is different for the trials with rotation of the head to the right (*i = hr*) and to the left (*i = hl*). The parameters of the lines were used to quantify the following variables:

• The *accuracy (Acc)*, that is average response-target distance, was represented by the average absolute distance between the regression lines and the line passing through the targets position (vertical gray lines in Figure 2A).
• The *Aubert-Müller effect (AMe)*, corresponding to the global response bias due to the lateral neck flexion (Guerraz et al., 1998), was quantified as half of the algebraic distance between the intersection point of the two regression lines with the vertical axis: *AMe* = (*q_*hl*_ - q_*hr*_*)/2.
• The *distortion (Dist)*, representing possible over/under-estimation of the distance between two targets’ orientation (McIntyre and Lipshits, 2008), is represented by the angle between the regression lines and the line passing through the targets’ orientations: *Dist* = atan(*m*)-45° (double arcs in Figure 2A). Positive and negative values of *Dist* correspond to a global over- and under- estimation of the angular distances, respectively.

### Sensory Weighting Quantification

To quantify the specific effect of the sensory conflict in each condition we linearly interpolated the responses of the conflict-trials with right and left neck flexion constraining the lines to be parallel to regression lines of the no-conflict-trials (see Figure 2B). This procedure provides the responses-axis intercepts for the conflict trials. Subtracting to these parameters the corresponding values in the no-conflict-trials we obtain the average deviations of the response due to the tilt of the visual scene: Δq_*r,hi*_. In order to convert the response deviation into the percentage weight given to visual information, we computed, for each conflict trial, the virtual displacement of the target expected if only visual information was used to code its orientation, which corresponds to *t - head_angle × 0.6*. We linearly interpolated these theoretical responses for right and left neck flexion separately, constraining the lines to be parallel to the one joining the targets (*m* = 1) and we obtained the response-axis intercepts (see Figure 2B). Subtracting from these parameters the intercept of the line joining the target in the no-conflict trials (*q* = 0), we obtain the average target deviation expected in case of fully visual encoding of their orientation: *Δq_*t,hi*_.* The percentage weight given to the visual information, ω_*V*_, can be then computed as it follows:


(2) $$\omega_{V}=\frac{1}{2}\,\sum_{i=l,r}\frac{\Delta \,q_{r,h\,i}}{\Delta \,q_{t,h\,i}}\cdot 100\%$$

### Statistical Analysis

For each experiment, we assessed the effect of the subject posture on the subject performances by performing mixed model ANOVAs on the *AMe*, *Dist*, *Acc*, *RMSd*, and *ω_*V*_* dependent variables, with the *Posture* (Seated, Supine) and *Order* (Seated-First and Supine-First) as within- and between- subjects independent variable, respectively. No between-experiment comparisons were performed, because they do not correspond to the goal of this study. Since we performed three distinct experiments, we applied a Bonferroni correction (*n* = 3) to the resulting p-values to reduce the probability of type I errors (false positive). Therefore, in the following, *p* < 0.05/3 (≃0.0167), *p* < 0.01/3 (≃0.0033), and *p* < 0.001/3 (≃0.00033) will be indicated with “*,” “^**^,” “^***^,” respectively. For the straight-neck experiment, we specifically wanted to test the “*Gravity Hp*,” that is whether the Supine position increased the subjects’ variable and constant errors. We therefore performed one-tail Student’s *t*-tests on *RMSd* and *Acc*. Since the subjects did not rotate their head, no conflict could be generated and no quantification of the sensory weighting was possible. All statistical analyses were performed using the Statistica 8 software (Statsoft, SCR_014213).

### Optimal Integration of Non-independent Sensory Signals Based on the Maximum Likelihood Principle

**FIGURE 3:**
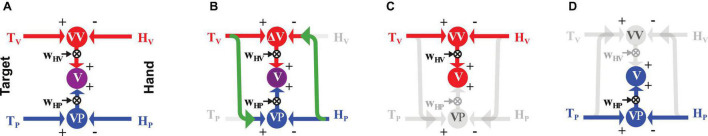
Concurrent Model of multisensory integration. **(A)** Graphical representation of the sensory information flow when the target, T, to be reached (on the left) and the hand, H, used to perform the movement (on the right) can be both sensed through vision, V (red), and proprioception, P (blue). ΔV and ΔP represent the concurrent representations in the visual and proprioceptive space of the movement to be performed to reach the target. The weights W_Δ*V*_ and W_Δ*P*_ (see Equation 3) allow one to optimally combine the concurrent representations and maximize the precision of the final motor vector estimation (Δ). **(B)** Application of the model to the cross-modal task of reaching a visual target with an unseen hand. Missing sensory cues are gray. The green arrows represent cross-modal transformations, that is, the encoding of an information coming from the visual system in the reference frame associated to the proprioceptive sensory system, V→P, or vice-versa, P→V. **(C,D)** Model application to uni-modal visual and proprioceptive tasks, respectively, where no cross-modal transformations are predicted.

In order to quantify the predictions associated with the *Gravity* and *Neck* Hypotheses and compare them with the experimental results, we apply our *Concurrent Model* of optimal sensory integration (Tagliabue and McIntyre, 2011, 2014) to describe the information flow associated with the Seated and Supine postures for each of the three experiments. An illustration of the general model structure is reported in Figure 3A.

This model is based on the assumption that the target and hand position are compared in the visual and proprioceptive space concurrently (ΔV and ΔP) and then these two parallel comparisons are combined based on the Maximum Likelihood Principle (Ernst and Banks, 2002). From this optimality principle it follows that the relative weight, W_ΔV_ and W_ΔP_, given to each comparison depends on their variance $\sigma_{△\,V}^{2}$ and $\sigma_{△\,P}^{2}$ as it follows:


$$W_{△\,V}=\frac{\sigma_{△\,P}^{2}-c\,o\,v\,\left(△\,V,△\,P\right)}{\sigma_{△\,V}^{2}+\sigma_{△\,P}^{2}-2\,c\,o\,v\,\left(△\,V,△\,P\right)}$$

(3) $$W_{△\,P}=\frac{\sigma_{△\,V}^{2}-c\,o\,v\,\left(△\,V,△\,P\right)}{\sigma_{△\,V}^{2}+\sigma_{△\,P}^{2}-2\,c\,o\,v\,\left(△\,V,△\,P\right)}$$

which corresponds to the minimal achievable variance of motor vector estimation Δ


(4) $$\sigma_{△}^{2}=\frac{\sigma_{△\,V}^{2}\,\sigma_{△\,P}^{2}-c\,o\,v\,{\left(△\,V,△\,P\right)}^{2}}{\sigma_{△\,V}^{2}+\sigma_{△\,P}^{2}-2\,c\,o\,v\,\left(△\,V,△\,P\right)}$$

In Equations 3 and 4 the covariance between ΔV and ΔP, *cov(ΔV,ΔP)*, is used to take into account the situations in which the two concurrent comparisons are not fully independent (Tagliabue and McIntyre, 2013). The application of MLP to multi-sensory integration therefore assumes that the brain can estimate the variability of the signals to be combined ($\sigma_{△\,V}^{2}$ and $\sigma_{△\,P}^{2}$) and to which extent they are independent (*cov*(△*V*,△*P*)). Although it is not clear whether, and how, the brain would actually estimate these specific parameters, perceptive and behavioral studies have shown that human sensory weighting is clearly modulated by signals’ variability as predicted by the MLP (Ernst and Banks, 2002) and that performances cannot be improved by combining two fully dependent signals (Tagliabue and McIntyre, 2013-2014), as expected if their covariance is taken into account.

For the cross-modal task without head rotation (Figure 3B), the model predicts a reconstruction of the proprioceptive target representation from the visual information and of a visual hand representation from the proprioceptive feedback (green arrows). These cross-modal transformations, which introduce additional errors, are associated to specific variance terms $\sigma_{V\to P}^{2}$ and $\sigma_{P\to V}^{2}$, and, as show in section 1 of Supplementary Material, Equations 3 and 4 become:


$$W_{△\,V}=\frac{\sigma_{V\to P}^{2}}{\sigma_{V\to P}^{2}+\sigma_{P\to V}^{2}}\;W_{△\,P}=\frac{\sigma_{P\to V}^{2}}{\sigma_{V\to P}^{2}+\sigma_{P\to V}^{2}}$$

(5) $$\sigma_{△}^{2}=\sigma_{T_{V}}^{2}+\sigma_{H_{P}}^{2}+\frac{\sigma_{V\to P}^{2}\,\sigma_{P\to V}^{2}}{\sigma_{V\to P}^{2}+\sigma_{P\to V}^{2}}$$

As illustrated in Figures 3C,D, the model predicts no cross-modal reconstructions for the unimodal tasks (Tagliabue and McIntyre, 2013): in these tasks, the direct comparison between the available information about the target and the hand fully covaries with any comparison reconstructed from the available cues. From equation 4 it follows that the reconstruction of concurrent comparisons cannot improve the precision of Δ and using equations 3 it results that the predicted sensory weights and the motor vector variance are:


(6) $$W_{△\,V}=1\;W_{△\,P}=0\;\sigma_{△}^{2}=\sigma_{T_{V}}^{2}+\sigma_{H_{V}}^{2}$$

(7) $$W_{△\,V}=0\;W_{△\,P}=1\;\sigma_{△}^{2}=\sigma_{T_{P}}^{2}+\sigma_{H_{P}}^{2}$$

for the visual and proprioceptive task, respectively.

For all tasks, once the motor vector is estimated, the motor system generates the muscle activations necessary to displace the hand in the defined direction and distance. This step introduces some additional noise, that we will call motor noise, $\sigma_{m}^{2}$, so that the variance of the movement execution is $\sigma_{M\,E}^{2}=\sigma_{△}^{2}+\sigma_{m}^{2}$. There might be additional factors, as the concentration and fatigue levels of the subject, that can contribute to the movement execution variability. For sake of simplicity, the present version of the model does not include them separately and they are all combined together in the $\sigma_{m}^{2}$ term.

To simulate the effect on the information processing of head inclination with respect to gravity, or of the neck flexion, in these three tasks, the variance, $\sigma_{N}^{2}$, is added to the $\sigma_{V\to P}^{2}$, $\sigma_{P\to V}^{2}$ terms. This extra noise is added to the cross-modal sensory transformations performed with the neck flexed, with neck muscle acting against gravity or with the head misaligned with respect to gravity, depending on the hypothesis to be tested.

In order to test which hypothesis, between the “Neck1,” “Neck2,” and “Gravity,” better predicts the experimental results, we compare the observed effect of posture on the subjects’ responses’ variability, $D\,M\,S\,d=R\,M\,S\,d_{S\,u\,p\,i\,n\,e}^{2}-R\,M\,S\,d_{S\,e\,a\,t\,e\,d}^{2}$, and on the response deviation due to visual scene rotation, *D*ω_*V*_=ω_*V*,*Supine*_−ω_*V*,*Seated*_, with corresponding parameters of the model: the difference between the Supine and Seated posture predicted by the model for the movement execution variability, $D\,\sigma_{M\,E}^{2}=\sigma_{M\,E,S\,u\,p\,i\,n\,e}^{2}-\sigma_{M\,E,S\,e\,a\,t\,e\,d}^{2}$, and for the weight associated with visual representation of the task, *DW*_△*V*_=*W*_△*V*,*Supine*_−*W*_△*V*,*Seated*_.

As shown in Supplementary Material (sections 3 and 4), the theoretical predictions depend only on two main parameters: the variance associated to the cross-modal sensory transformation, $\sigma_{P↔V}^{2}$, and to the noise added to these transformations when performed with the head misaligned with respect to gravity and/or the body,$\sigma_{N}^{2}$. In order to reduce even further the degrees of freedom of the model, and thus the possibility of overfitting the experimental data, the value of $\sigma_{P↔V}^{2}$ is set to 23.19°^2^; a value that is computed from the results of Tagliabue and McIntyre (2011) in section 4.2 of Supplementary Material. To statistically test whether the predictions of the various hypotheses differed from the experimental data, a multivariate Hotelling’s *T*^2^ test is performed with six dependent variables (*D*ω_*V*_ and *DMSd* for each of the three experiments) and the six corresponding model predictions (*DW*_*△V*_ and $D\,\sigma_{M\,E}^{2}$) as reference values.

## Results

**FIGURE 4:**
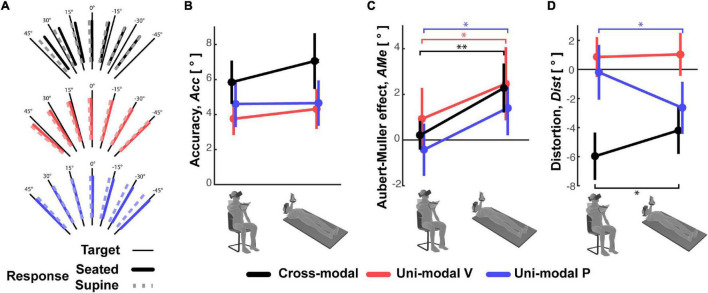
Average subject responses. **(A)** For the three experiments, the mean orientation of the subjects’ responses to each target orientation is represented for the Seated and Supine conditions. The three parameters representing **(B)** the response accuracy, **(C)** Aubert-Müller effects, that is the global bias of the responses due to lateral neck flexion. Positive values correspond to deviations toward the head direction. **(D)** Response distortions are reported for the Seated and Supine conditions of the three experiments. Vertical whiskers correspond to 95% confidence intervals. * and ^**^ represent *p* < 0.05/3 and *p* < 0.01/3, respectively, and their color represents the experiment to which they refer.

The subjects’ average responses in the three main experiments (Cross-modal, Unimodal Visual and Unimodal Proprioceptive tasks) for the two tested postures (Seated and Supine) are depicted in Figure 4A, where specific deviations of the responses away from the target can be seen for each task and each posture. The statistical analyses show that none of the analyzed parameters were significantly affected by the posture *Order* and that the *Order* did not significantly interact with the *Posture* effect. Neither did *Posture* appear to have had a significant effect on the average error (accuracy) in any of three experiments (Figure 4B). More detailed analyses of the pattern of errors, however, reveal some specific effects of *Posture* (see statistics reported on Table 1): the global response deviation in relation with the lateral neck flexion, close to zero in the Seated condition, significantly increased in all three experiments when the subjects were Supine (Aubert-Müller effect in Figure 4C). The effect of posture on the perceptive distortion appears to have differed among the three experiments (Figure 4D): a significant modulation, but in opposite directions, for cross-modal and unimodal proprioceptive tasks and no difference for the unimodal visual experiment. In conclusion, subjects’ posture appears to affect some specific aspect of the average response patterns, but the average error (accuracy) does not significantly change when supine.

**TABLE 1:** For each of the experiments (cross-modal, V-P; unimodal visual V-V; unimodal proprioceptive, P-P) the ANOVA main effect of Posture, posture Order and the interaction between these two factors are reported for the Aubert-Müller effect, AMe, the response distortion, Dist, accuracy, Acc, and variability, RMSd, as well as for the relative weight associated to visual information, ω_*V*_.

		Effects		
Exp	Param.	Posture	Order	Posture x Order
	AMe	*F*_(1,16)_ = 12.7, ***p* = 0.0026**	*F*_(1,16)_ = 0.18, *p* = 0.67	*F*_(1,16)_ = 0.01, *p* = 0.89
	Dist	*F*_(1,16)_ = 10.6, ***p* = 0.0049**	*F*_(1,16)_ = 0.23, *p* = 0.63	*F*_(1,16)_ = 0.61, *p* = 0.44
V-P	Acc	*F*_(1,16)_ = 0.97, *p* = 0.34	*F*_(1,16)_ = 0.97, *p* = 0.33	*F*_(1,16)_ = 0.34, *p* = 0.57
	RMSd	*F*_(1,16)_ = 15.3, ***p* = 0.0012**	*F*_(1,16)_ = 0.01, *p* = 0.91	*F*_(1,16)_ = 1.41, *p* = 0.25
	ω_*V*_	*F*_(1,16)_ = 23.9, ***p* = 16^∙^10^–5^**	*F*_(1,16)_ = 0.00, *p* = 0.97	*F*_(1,16)_ = 0.57, *p* = 0.46
	AMe	*F*_(1,16)_ = 9.16, ***p* = 0.0080**	*F*_(1,16)_ = 0.19, *p* = 0.67	*F*_(1,16)_ = 2.91, *p* = 0.11
	Dist	*F*_(1,16)_ = 0.01, *p* = 0.93	*F*_(1,16)_ = 0.20, *p* = 0.65	*F*_(1,16)_ = 2.49, *p* = 0.13
V-V	Acc	*F*_(1,16)_ = 1.63, *p* = 0.22	*F*_(1,16)_ = 0.42, *p* = 0.52	*F*_(1,16)_ = 0.86, *p* = 0.37
	RMSd	*F*_(1,16)_ = 0.10, *p* = 0.76	*F*_(1,16)_ = 0.07, *p* = 0.79	*F*_(1,16)_ = 0.85, *p* = 0.37
	ω_*V*_	*F*_(1,16)_ = 2.36, *p* = 0.14	*F*_(1,16)_ = 0.25, *p* = 0.62	*F*_(1,16)_ = 3.54, *p* = 0.08
	AMe	*F*_(1,16)_ = 10.9, ***p* = 0.0044**	*F*_(1,16)_ = 0.98, *p* = 0.34	*F*_(1,16)_ = 2.11, *p* = 0.16
	Dist	*F*_(1,16)_ = 10.7, ***p* = 0.0048**	*F*_(1,16)_ = 0.01, *p* = 0.92	*F*_(1,16)_ = 6.93, *p* = 0.018
P-P	Acc	*F*_(1,16)_ = 0.01, *p* = 0.93	*F*_(1,16)_ = 4.94, *p* = 0.04	*F*_(1,16)_ = 0.04, *p* = 0.83
	RMSd	*F*_(1,16)_ = 0.85, *p* = 0.37	*F*_(1,16)_ = 2.89, *p* = 0.11	*F*_(1,16)_ = 0.98, *p* = 0.33
	ω_*V*_	*F*_(1,16)_ = 0.02, *p* = 0.89	*F*_(1,16)_ = 0.71, *p* = 0.41	*F*_(1,16)_ = 4.31, *p* = 0.054

*The significant results after the Bonferroni correction (p < 0.05/3) are reported in bold fonts.*

**FIGURE 5:**
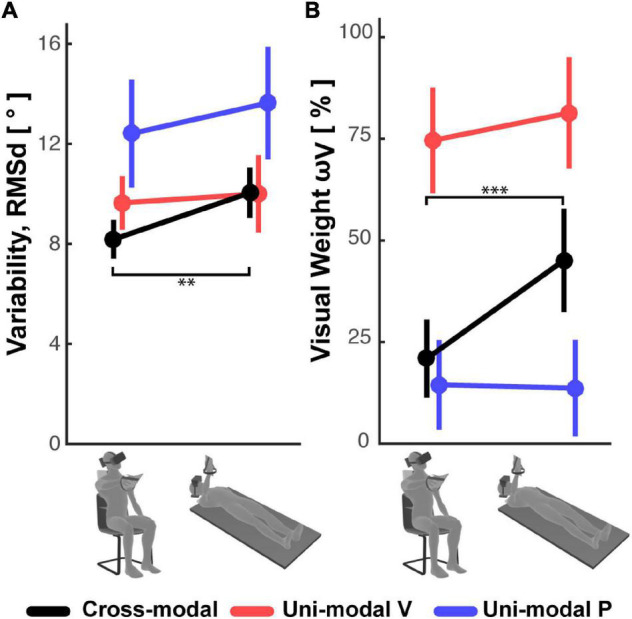
**(A)** Subjects’ response variability and **(B)** visual weight observed in the Seated and Supine conditions for the cross-modal and the two uni-modal (visual and proprioceptive) experiments. Vertical whiskers correspond to 95% confidence intervals. ^**^ and ^***^ represent *p* < 0.01/3 and *p* < 0.001/3, respectively, and their color represents the experiment to which they refer.

On the other hand, the variability of the responses RMSd, reported in Figure 5A, appears to have been affected by the subject’s posture: in the cross-modal experiment the subjects were significantly less precise when supine, but this was not the case in the unimodal visual and proprioceptive experiments. The change, or lack thereof, in response variability was accompanied by a similar modulation of the sensory weighting shown in Figure 5B: only in the cross-modal task did the visual weight significantly increase in the supine posture.

Overall, these results suggest that the use of sensory information during the cross-modal paradigm differs from that of unimodal tasks, and that this weighted processing is significantly affected by posture.

### Analysis of Between-Subjects Differences

To go beyond average responses, we then assessed whether inter-individual variability can provide more insight on the sensory processing underlying the responses observed in the three experiments.

For the Seated condition of the unimodal visual and proprioceptive experiments, the concurrent model predicts, respectively, a negative and positive correlation between the visual weighting and the variability of the motor vector estimation. In fact, a visual weight smaller than 100% in the V-V task, or the larger than 0% in the P-P task, would both correspond to suboptimal solutions and thus to an increase of the variability of the motor vector estimation (see Supplementary Material, section 2). The correlation between visual weighting and the variability of the motor vector estimation measured in inter-individuals is reported in Table 2.

**TABLE 2:** Coefficient of correlation R (and associated p-value) between the variability, RMSd, and visual dependency, ω_*V*_, in the Seated condition of the three experiments (Exp).

Exp	*R*	*p*
V-P	0.11	0.65
V-V	−0.41	0.09
P-P	0.17	0.51

Although not statistically significant, the tendency to a negative correlation in the unimodal visual task reported in Table 2, is consistent with the model prediction, while the absence of correlation in the P-P experiment is not. This could be due to a significant contribution of the motor noise to *RMSd* in this task, because both memorization and response require active hand movements. Motor noise affects the response variability but not the sensory weight, thus it might hide an existing correlation between the variability of motor vector estimation and the sensory weighting. The potential influence of motor noise is supported by the fact that the expected correlation seems to exist for the V-V task, where the motor component should be irrelevant.

For the V-P task no clear correlation between *ω_*V*_* and *RMSd* is to be expected, because, as shown in Equation 5, the sensory weight theoretically depends only on the noise attributed to the cross-modal sensory transformations, whilst the response variability depends also on the subject’s visual and proprioceptive acuity. Moreover, motor noise could play a role, as in the P-P task.

**FIGURE 6:**
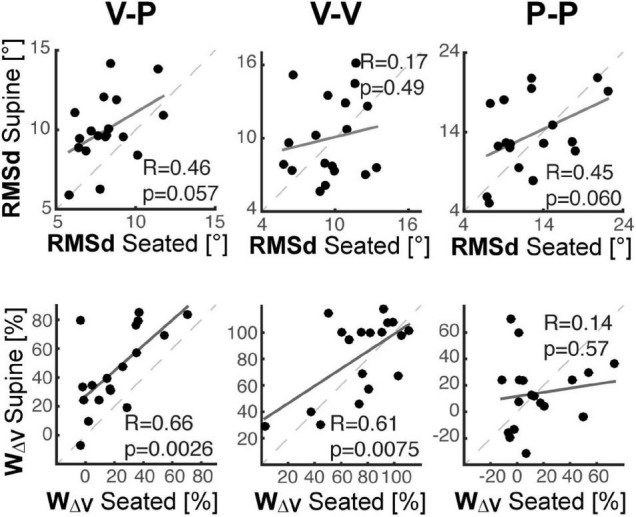
Inter-individual analyses. For each of the three experiments cross-modal (left), unimodal visual (middle) and unimodal proprioceptive (right), individual performance in the Supine condition are shown as a function of the performance in the Seated condition, in terms of response variability (top row) and visual dependency (bottom row). Dashed lines correspond to the identity line. Solid lines correspond to the linear interpolation of the data. “R” is the coefficient of correlation between the Seated and Supine data and “p” represents the corresponding statistical significance.

In order to understand whether between-subject differences while seated would affect an individual’s performance when supine, we evaluated the correlation between the individual performance in the Seated and Supine conditions. As shown in Figure 6, we evaluated the performance in terms of response variability, *RMSd*, and visual weight, *ω_*V*_*.

The top part of Figure 6 shows that the ranking of the subject in terms of response precision in the Seated condition tends to be preserved when Supine, but only in the tasks with relevant proprioceptive and motor components (V-P and P-P). Consistent with the results of Table 2, this finding suggests that the individual motor noise contributes to the observed response variability and tends to be preserved between postures. The bottom part of Figure 6 show that in the tasks with a relevant visual component (V-P, V-V), the subjects that are most visuo-(in)dependent when seated, remain the most visuo-(in)dependent when supine. These correlations suggest that, although different levels of visual-dependency can be observed among the subjects, their visual-dependency ranking was not altered by posture. It follows that the effect of the postural change in the cross-modal task was quite consistent among all of participants.

### Model Predictions

**FIGURE 7:**
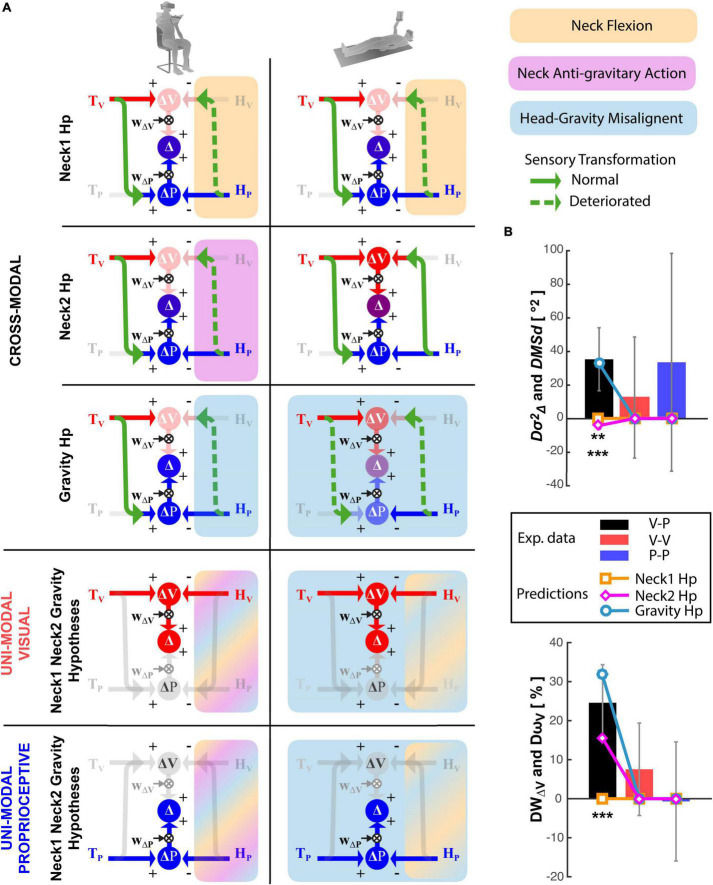
Model predictions. **(A)** Graphical representation of the sensory information flow in the Seated (left) and Supine (right) conditions for the cross-modal, unimodal visual and unimodal proprioceptive experiments. For the cross-modal task the predictions for the Neck1, Neck2, and Gravity hypotheses are represented separately. For the unimodal visual and proprioceptive tasks, the three hypotheses are identical and thus represented together. The model structures and the graphical conventions are the same as in Figure 3. In addition, dashed green arrows represent perturbed cross-modal sensory transformations; faded arrows and circles are associated with a noisy information. For each tested theory the colored rectangular areas include the cross-modal transformations perturbed by the hypothesized disrupting factor: orange, violet and cyan represent the neck flexion, the neck muscles action against gravity and the head-gravity misalignment, respectively. Since for the unimodal tasks the three hypotheses are represented together, multicolor areas illustrate the cross-modal transformations affected by more the one disrupting factor. **(B)** Comparison between the experimental results and the predictions of the three hypotheses, in terms of modulation of the response variance (upper panel) and visual weight (lower panel) due to postural change (Supine-Seated). Vertical whiskers represent the 95% confidence interval of the experimental data. ** and *** represent statistical difference (*p* < 0.01 and *p* < 0.001) between the model predictions and the experiments results for each experiment and each parameter separately. The color of the stars indicates the tested hypothesis.

Figure 7A graphically represents the model predictions associated with the hypotheses that the lateral neck flexion per se (Neck1 Hp), the increase of the noise in the neck muscles-spindles (Neck2 Hp) or the head misalignment with respect to gravity (Gravity Hp), interferes with the ability to perform cross-modal transformation (detailed model equations are presented in Supplementary Material, section 3). Their quantitative comparison with the experimental results is shown in Figure 7B in terms of differences between the Seated and Supine condition. Focusing these predictions on the effect of the postural change has two main advantages: first, it compensates for a possible role of individual motor precision or sensory acuity that, as we have shown above, might increase between-subject variability. Second, it simplifies the model by allowing a significant reduction of the number of parameters estimated.

Figure 7B show that the “Neck1 Hp,” which predicts no changes between Seated and Supine postures for all three, Cross-Modal, Unimodal Visual and Unimodal Proprioceptive tasks, is significantly different from the experimental observations [Hotelling’s test: *T*^2^ = 93.0, *F*_(6,12)_ = 10.9, *p* = 0.0003]. The “Neck2 Hp” prediction also significantly differs from the experimental observations [Hotelling’s test: *T*^2^ = 34.93 *F*_(6,12)_ = 4.11, *p* = 0.017]. Indeed, although this hypothesis appears to better match the increase of the visual weight when supine, it cannot account for the increase in response variability; since in the Supine posture the neck muscles never act against gravity the model must predict a decrease of the response variability with respect to task performed with the Seated posture, which require a neck muscles’ activation during the response phase to support the tilted head.

“Gravity Hp” appears to well capture the fact that the Supine posture increases both the response variability and the visual weight in the cross-modal task only [Hotelling’s test: *T*^2^ = 9.65, *F*_(6,12)_ = 1.13, *p* = 0.40]. The matching between the Gravity Hp prediction and the experimental data is obtained with $\sigma_{N}^{2}=81^{2}$, which means that the variance associated with the cross-modal transformation would increase by about 3.5 times when the head is not aligned with gravity.

### Straight-Neck Experiment

**FIGURE 8:**
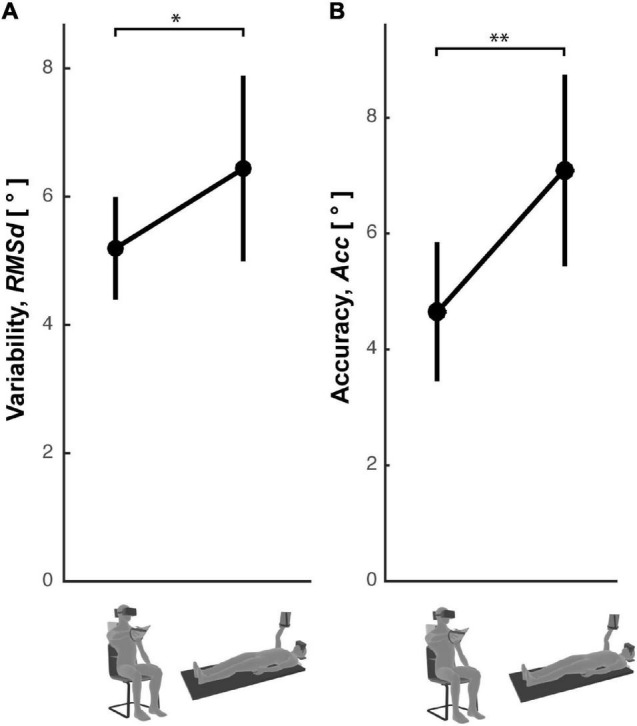
Response errors in the Straight Neck Experiment. Subjects’ response **(A)** variability and **(B)** accuracy observed in the Seated and Supine conditions for the cross-modal task without lateral neck flexions. Vertical whiskers correspond to 95% confidence intervals. * and ** represent *p* < 0.05 and *p* < 0.01, respectively.

To confirm the role of the head-gravity alignment on the visuo-proprioceptive transformations (experimental results and the model prediction of Figure 7) the precision and the accuracy of the subjects’ responses was compared between the Seated and Supine conditions of a cross-modal task performed without lateral neck movements. Figure 8 shows that, as for the main Cross-Modal Experiment, when supine the subjects are significantly less precise [one-tailed *t*-test: *t*_(11)_ = 3.42, *p* = 0.04] and less accurate [one-tailed *t*-test: *t*_(11)_ = 2.79, *p* = 0.009] than when seated.

## Discussion

We have performed experiments to try to understand why lateral neck flexions appear to interfere with the visuo-proprioceptive transformations used during reaching/grasping movements (Burns and Blohm, 2010; Tagliabue and McIntyre, 2011, 2014; Tagliabue et al., 2013). This type of cross-modal transformation consists of encoding retinal visual signals into a proprioceptive joint space and, vice-versa, encoding the position/orientation of the hand sensed through joint proprioception in a visual space.

Our first working hypothesis was that neck flexion might perturb the sensory information coming from the eye-hand kinematic chain, which can be used for computing the cross-modal transformation (Sabes, 2011). The lateral neck flexion interference could have two main origins: the rarity of performing eye-hand coordination tasks with such neck configuration (Neck1 Hp) or degradation of the proprioceptive neck information due to the muscle effort necessary to sustain the head’s weight (Neck2 Hp). “Neck1 Hp” is related to the difficulty of interpreting correctly the “unusual” sensory signals coming from the flexed neck. As observed for different tasks, motor performance appears indeed to correlate with the relative incidence of the type of movement during everyday life (Howard et al., 2009). “Neck2 Hp” is based on the signal-dependent nature of noisiness of the neck muscles spindles (Abedi Khoozani and Blohm, 2018). An alternative hypothesis, one that does not involve the eye-hand kinematic chain, was that head misalignment with respect to gravity, and not lateral neck flexion, would mainly interfere with visuo-proprioceptive transformations (Gravity Hp). This hypothesis is based on the fundamental role that gravity would have in reciprocal calibration of the retinal and proprioceptive reference frame (Paillard, 1991).

To test which of these hypotheses better describe the actual functioning of the human central nervous system (CNS) we asked volunteers to perform a virtual-reality task requiring cross-modal transformations, i.e., matching with an unseen hand a memorized visual target orientation, as to grab it, after a lateral neck flexion. The subjects performed this task both in a Seated and Supine position.

The expected effect of changing posture is very different for the three hypotheses. To try to formalize and quantify these predictions we applied an optimal theory of multi-sensory integration to the above-described task. This statistical model, in which the task is concurrently represented in the visual and proprioceptive space (Tagliabue and McIntyre, 2008, 2011, 2012, 2013, 2014; McGuire and Sabes, 2009; Tagliabue et al., 2013; Arnoux et al., 2017; Bernard-Espina et al., 2021) allowed to compute the effects of changing posture in terms of subjects’ responses variability and in terms of the relative importance given to the visual and proprioceptive encoding of the information.

The model results show that the “Neck1 Hp” predicts no significant changes in subject precision nor in sensory weighting, because the lateral neck flexion is the same in the two postural conditions. If the “Neck2 Hp” is correct a decrease of the response variability and an increase of the importance given to visual encoding is to be expected, because when supine a special head support always sustained the head, reducing the neck muscles activation, and hence the neck proprioceptive noise. The “Gravity Hp” predicts an increase of both response variability and weight associated to visual space, because when supine the subject head is always misaligned with respect to gravity, continuously perturbing cross-modal transformations. The results of the “Cross-Modal Experiment” show a significant increase of the response variability and visual weight when supine, so that the “Gravity Hp” prediction is the closest to the experimental observations. With the “Neck-Straight Experiment,” which does not involve lateral head rotations, we were able to disentangle even further the role of gravitational afferences from those generated by neck movements, such as neck muscle spindles and semi-circular canals signals. The persistence, in this experiment as in the task with head rotations, of an increase of subject errors in the supine posture confirms and reinforce the importance of the gravity-head alignment. Overall, these results clearly support the hypothesis of a fundamental role of gravity in the ability of performing cross-modal transformations. More precisely, these findings are consistent with the idea that a misalignment of the head with respect to gravity interferes with the ability of performing cross-modal transformations, that is the encoding a visual information in the proprioceptive space and vice-versa.

Although the present results support the central role of the external gravitational reference, a role of the neck and of the rest of eye-hand kinematic chain, which is associated with an egocentric processing of the information, should not be fully discarded. We have indeed already reported evidence supporting the coexistence of ego- and exo-centric information processes (Tagliabue and McIntyre, 2012, 2014). Moreover, a role of the visual vertical in the ability to perform cross-modal sensory transformations cannot be excluded, as it has been shown that the vertical direction perception is a highly multisensory process, with gravity, body and scene information interacting (Dyde et al., 2006).

The posture effect on the cross-modal transformations reported here, however, is ascribable to gravitational signals, because in all used experimental paradigms the head/body axis information and the visual information contributing to the vertical perception were identical in the seated and supine condition and the only factor that changed was the misalignment with respect to the gravitational vector.

To be able to exclude the hypothesis that the observed effect of the posture in the cross-modal task could be ascribed to a degradation of the visual or proprioceptive acuity *per se* and not of the sensory transformations, we added two control experiments in which the subjects performed visual and proprioceptive tasks not requiring sensory transformations. The lack of significant differences between the seated and supine condition in terms of response variability and sensory weighting in these uni-modal experiments suggests that the head misalignment with respect to gravity does not significantly alter the unimodal sensory precision *per se*, and thus supports the idea of a specific effect of posture/gravity on the sensory transformations. The different effect of the posture on the response precision between the cross-modal and unimodal tasks is perfectly in line with the results of the orientation reproduction experiment of McIntyre and Lipshits (2008). They showed indeed that laterally tilting the whole body of subjects by 22.5° clearly increases their response errors in a cross-modal (haptic-visual) task, and not so in two unimodal tasks (visual-visual and haptic-haptic). The consistency with the present results also suggests that the head tilt effects are independent of the tilt axis (pitch or roll).

In our three first experiments we observed that posture also influences some features of the average pattern of subjects’ responses. Although our theoretical framework does not provide predictions on this aspect of the subjects’ performance, it is interesting to note that the response shifts due to the lateral neck flexion (Aubert-Müller effect) significantly increased when supine, in all three experiments. This result suggests that gravity direction would also contribute to the encoding of the target and response orientation, no matter the modality of the information. This is consistent with Darling and Gilchrist (1991) study on hand orientation reproduction tasks showing that gravitational information influences the encoding of the hand roll. Similarly, the disappearance of the oblique effect when the subject’ whole body is laterally tilted in purely visual (McIntyre et al., 2001) and cross-modal (McIntyre and Lipshits, 2008) orientation reproduction tasks was interpreted as an evidence of the use of gravity as a reference to encode orientation cues. In addition to its role in perception, gravity was shown to contribute also to motor encoding, since lateral tilts affected the perception of hand movements direction (Darling et al., 2008) and the control of eye saccades (Pelt et al., 2005).

### Inter-Individual Differences

The analyses of the between-subjects differences suggest that the effect of the head-gravity misalignments on cross-modal transformations is quite robust, since it does not appear to depend on individual characteristics such as visual dependency or precision, which can vary significantly between subjects. The observed inter-subject variability in the Seated condition also suggests that not all subjects perform optimally, in the “Maximum Likelihood” sense (Ernst and Banks, 2002), that is, some subjects sub-optimally combine the visual and proprioceptive representations of the task. As expected, however, those subjects who deviate from the theoretical optimal sensory weighting tends to show larger level of variability.

Lastly, the inter-subject analyses also suggest that the noise of the motor component of the task, which can be different between participants, might represent a relevant part of the performance variability. These observations confirm the rationale of basing our conclusions on within-subject comparisons.

### Vestibular Pathways to Cortical Networks Involved in Visuo-Proprioceptive Transformations

The present section aims at discussing whether the behavioral findings reported here are compatible with the current knowledge about the anatomy and physiology of the central nervous system. First, the brain areas involved in visuo-proprioceptive transformations will be presented. Second, it will be discussed how the signals related to head orientation with respect to gravity might interact with these brain areas and hence with the cross-modal processing.

The idea that the brain performs cross-modal transformations is supported by several electrophysiological and brain imaging studies. For instance, the encoding of visual stimuli in somatosensory space is consistent with the observation that brain regions such as the somatosensory areas (S) and Broadman’s Area 5 (BA5), which are known to encode the hand grasping configuration and the position of tactile stimulation in the peripersonal space (Koch and Fuster, 1989; Deshpande et al., 2008; Lacey et al., 2009), are activated also by visual stimuli such as images of glossy and rough surfaces, which a have a strong “tactile content” (Sun et al., 2016), and by images of familiar manipulable objects (Vingerhoets, 2008). Similarly, the encoding of haptic/proprioceptive information in visual space is fully compatible with the finding that the visual area in the Lateral Occipital Complex, called LOtv, is activated not only by 3D objects images (Moore and Engel, 2001), but also when sensing familiar objects with the hand (Deshpande et al., 2008; Lacey et al., 2009).

A brain area which appears to be a good candidate for performing cross-modal transformations is the Intra-Parietal Sulcus (IPS) which has been shown to have neural activation compatible with the computation of visuo-tactile transformations in monkey (Avillac et al., 2005) and which is known to be involved in the visuo-motor transformations performed during grasp movements (McGuire and Sabes, 2011; Janssen and Scherberger, 2015). Monkey experiments have shown that, in this brain area, the information can be reencoded from the retinal space to the somatosensory space, and vice-versa, thanks to recurrent basis function neural networks (Pouget et al., 2002) which would use the sensory signals relative to the eye-body kinematic chain to “connect” the two sensory spaces. In humans, the Anterior part of IPS is strongly activated when comparing visual to haptic objects, and vice-versa (Grefkes et al., 2002) or when reaching a visual target without visual feedback of the hand (Beurze et al., 2010). Virtual lesions of this area through TMS interfere with visuo-tactile transformations, but not with uni-modal, visual and tactile, tasks (Buelte et al., 2008). The planning of cross-modal tasks, such as reach-and-grasp visual objects with an unseen hand, also appears affected by TMS of the anterior IPS (Verhagen et al., 2012).

Focusing on the main finding of the present study, one can ask through which neural pathway the head-gravity misalignment can affect the visuo-proprioceptive transformations occurring in the IPS. At the peripheral level, the information about the head orientation with respect to gravity is mainly provided by a complex integration of the signals from different areas of the otolithic organ (Chartrand et al., 2016) arising from both the left and right organs (Uchino and Kushiro, 2011). Semi-circular canal and neck proprioception, which are combined to otolithic information already at the level of the vestibular nuclei (Gdowski and McCrea, 2000; Dickman and Angelaki, 2002), can also contribute to the head orientation estimation. However, since in the Straight Neck Experiment the posture effect was also observed when no head rotations, nor neck flexions, occurred, we can conclude that the otolithic signals are sufficient to affect visuo-proprioceptive transformations. At the central level, it is known that the vestibular-otolithic information can reach the parietal cortex through the posterior vestibular thalamocortical pathway (Hitier et al., 2014; Cullen, 2019). Specific otolithic afferences have been indeed observed in the IPS: otolithic stimulations activate neurons of Ventral IPS in monkeys (Schlack et al., 2002; Chen et al., 2011), with half of the neurons in this area which receive vestibular inputs (Bremmer, 2005), and human fMRI studies also show IPS activations resulting from saccular stimulations (Miyamoto et al., 2007; Schlindwein et al., 2008). Electrical stimulations of the anterior-IPS have also been reported to elicit linear vestibular sensations in a patient (Blanke et al., 2000). Since head-gravity misalignment modulates the otolithic inputs and the otolithic system projects to the IPS, it is plausible that gravitational information would be integrated in the recurrent basis-function neural network of this brain areas (Pouget et al., 2002; Avillac et al., 2005) to “connect” the visual and the proprioceptive space. As a consequence, it is reasonable that an alteration of the otolithic gravitational input due to the head tilt can alter cross-modal transformations.

There are other neural structures involved in motor control, such as the cerebellum, that receive otolithic inputs (Büttner-Ennever, 1999), and could therefore contribute to the effect of the head-gravity misalignment observed here. However, the predictive functions of the cerebellum (Blakemore and Sirigu, 2003), which is fundamental for the control of rapid movements, probably plays only a marginal role in the slow, quasi-static, movements tested here.

### Otolithic Signal-Dependent Noise or Unusualness?

Once we have established that the head-gravity misalignment affects visuo-proprioceptive transformations and which neural circuits could be responsible for this phenomenon, the following question remains open: “How does tilting the head interfere with the cross-modal sensory processing?” At least two possible explanations exist: first, the unusualness of performing eye-hand coordination tasks with the head tilted; second, a possible signal-dependent increase of the otolithic noise with the head tilt.

Some studies have been able to correctly predict the effect of tilting the head on subjective vertical experiments by assuming that the noise of the otolithic signals linearly increases with the signal amplitude (Vrijer et al., 2008), hence the second hypothesis appears reasonable. To our knowledge, however, there are no electrophysiological studies clearly supporting the signal-dependent modulation of the otolithic noise (Fagerson and Barmack, 1995; Yu et al., 2012), therefore, the fact that unusual tilt of the head could interfere with cross-modal sensory transformations should not be “*a priori*” discarded. The “usualness effect” appears consistent with IPS recurrent neural networks functioning (Pouget et al., 2002) in which the synaptic weights necessary to perform visuo-proprioceptive transformations are learnt through experience. Since the upright position is largely the most common head orientation in our everyday life, it is possible that these neural networks become “optimized” for such head position and significantly less effective when otolithic afferences signal a head tilt for which we have a limited experience. A way to test this hypothesis could be to perform experiments on subjects that are in a tilted position, or in weightlessness, for a long period of time and see whether they can learn to perform cross-modal transformations as effectively as in the upright position, despite the altered or lacking otolithic signals.

## Conclusion and Perspectives

The results of the present study show the relevant role of the head-gravity alignment in the ability of performing visuo-proprioceptive transformations necessary to correctly reach and grasp objects. This finding suggests that the neural networks in the parietal cortex involved in the cross-modal processing of sensory information are more efficient when the otolithic afferences correspond to an upright head position.

This finding has interesting implications: for instance, the application of this idea to the clinical field suggests that vestibular pathologies might perturb not only equilibrium and eye movements, but also the eye-hand coordination, which is rarely assessed in these patients. Our findings might be beneficial also to healthy subjects, in that they can contribute to the ergonomic principles used when conceiving a new working station: avoiding visuo-manual tasks when the operator is tilted would indeed maximize their execution precision. Finally, there are potential space-related applications: the astronauts’ eye-hand coordination might be perturbed in weightlessness, because of the lack of the gravitational reference used for visuo-proprioceptive transformations. To prevent potential deterioration of performances in delicate visuo-manual tasks, as controlling robotic-arms or piloting space vehicles, specific training performed in “altered” posture could therefore be beneficial.

## Data Availability Statement

The raw data supporting the conclusions of this article will be made available by the authors, without undue reservation.

## Ethics Statement

The studies involving human participants were reviewed and approved by CER Université de Paris. The patients/participants provided their written informed consent to participate in this study.

## Author Contributions

MT conceived and supervised the experiments, performed the final data analysis, and wrote the first draft of the manuscript. JB-E performed the experiments and data analyses. DD developed the experimental setup and performed the experiments. All authors contributed to manuscript revisions, read and approved the submitted version.

## Conflict of Interest

The authors declare that the research was conducted in the absence of any commercial or financial relationships that could be construed as a potential conflict of interest.

## Publisher’s Note

All claims expressed in this article are solely those of the authors and do not necessarily represent those of their affiliated organizations, or those of the publisher, the editors and the reviewers. Any product that may be evaluated in this article, or claim that may be made by its manufacturer, is not guaranteed or endorsed by the publisher.
