# Supplementary material for: How Tilting the Head Interferes With Eye-Hand Coordination: The Role of Gravity in Visuo-Proprioceptive, Cross-Modal Sensory Transformations
Source: Front Integr Neurosci. 2022 Mar 10;16:788905. doi: 10.3389/fnint.2022.788905 (PMC8961421; doi:10.3389/fnint.2022.788905)
Supplement: Supplementary file 1 [file Data_Sheet_1.docx]

Supplementary Material

# Optimality in multisensory integration

The motor vector $\Delta$ is the weighted sum of the sensory estimates in the visual ($\Delta V$) and proprioceptive ($\Delta P$) modality, and its variance is:

| $\sigma_{\boldsymbol{\Delta}}^{2}={W_{\Delta P}}^{2}\sigma_{\Delta P}^{2}+{W_{\Delta V}}^{2}\sigma_{\Delta V}^{2}+2W_{\Delta P}W_{\Delta V}cov(\Delta P,\Delta V)$  where $W_{\Delta P}+W_{\Delta V}=1.$ | **(S1)** |
| --- | --- |

According to the Maximum Likelihood Principle (MLP), to minimize the motor vector’s variance $\sigma_{\boldsymbol{\Delta}}^{2}$, the sensory weights $W_{\Delta V}$ and $W_{\Delta P}$ are the following:

| $W_{\Delta V}=\frac{\sigma_{\Delta P}^{2}-cov(\Delta V,\Delta P)}{\sigma_{\Delta V}^{2}+\sigma_{\Delta P}^{2}-2cov(\Delta V,\Delta P)}$ , $W_{\Delta P}=\frac{\sigma_{\Delta V}^{2}-cov(\Delta V,\Delta P)}{\sigma_{\Delta V}^{2}+\sigma_{\Delta P}^{2}-2cov(\Delta V,\Delta P)}$ | **(S2)** |
| --- | --- |

The optimal solutions for the cross-modal task (V-P), unimodal visual task (V-V) and unimodal proprioceptive task (P-P) are described in the Manuscript (equations 5-7). For further details on the mathematical procedure, please refer to Supplementary Material of Bernard-Espina et al. (2021).

# Predictions for sub-optimal strategies

For the unimodal tasks, the MLP predicts that a visual weight smaller than 100% in the V-V task, or larger than 0% in the P-P task, would correspond to a suboptimal strategy, increasing the variance of the motor vector.

The experimental results suggest that not all subjects used the sensory information in an optimal fashion (see Manuscript, section “Analysis of between-subjects differences” in Results).

## Unimodal visual task (V-V)

For the V-V task, the variance associated with the visual and proprioceptive representations are the following:

| $\begin{matrix} \vert\mathrm{Target} & \vert\mathrm{Hand} \\ \sigma_{\Delta V}^{2}=\sigma_{T_{V}}^{2} & +\sigma_{H_{V}}^{2} \\ \sigma_{\Delta P}^{2}=\sigma_{T_{V}}^{2}+\sigma_{V\to P}^{2} & +\sigma_{H_{V}}^{2}+\sigma_{V\to P}^{2} \end{matrix}$ | **(S3)** |
| --- | --- |

Since there is no proprioceptive information, the proprioceptive representation of both the target and the hand need to be reconstructed from the visual information. Therefore, the covariance between visual and proprioceptive representations is:

| $cov\left( \Delta P,\Delta V \right)=\sigma_{T_{V}}^{2}+\sigma_{H_{V}}^{2}=\sigma_{\Delta V}^{2}$ | **(S4)** |
| --- | --- |

If we assume a generic, therefore possibly suboptimal, visual weigh W (with $W\in\left[ 0 1 \right]$), and replacing S3 and S4 in S1, we obtain:

| $\sigma_{\boldsymbol{\Delta}}^{2}=\sigma_{\Delta V}^{2}+{(1-W)}^{2}\left( 2\sigma_{V\to P}^{2} \right)$ | **(S5)** |
| --- | --- |

To complete this formulation, we must take into consideration the head tilt occurring during the response phase which induces a noise in the cross-modal sensory transformation $\sigma_{\boldsymbol{N}}^{2}$. Therefore, equation S5 becomes:

| $\sigma_{\boldsymbol{\Delta}}^{2}=\sigma_{\Delta V}^{2}+{(1-W)}^{2}\left( 2\sigma_{V\to P}^{2}\boldsymbol{+}\sigma_{\boldsymbol{N}}^{2} \right)$ | **(S6)** |
| --- | --- |

It follows that, if W is smaller than the optimal visual weight $W_{\Delta V}=1$ (equation 6 in the main text), the variability of the associated movement vector estimation is larger than the variability of the optimal MLP solution $\sigma_{\boldsymbol{\Delta}}^{2}=\sigma_{\Delta V}^{2}$ (Supplementary Figure 1). A negative correlation between *W* and $\sigma_{\boldsymbol{\Delta}}^{2}$ is hence to be expected.

## Unimodal proprioceptive task (P-P)

Similarly, for the P-P task, we obtain the following:

| $\sigma_{\boldsymbol{\Delta}}^{2}=\sigma_{\Delta P}^{2}+W^{2}\left( 2\sigma_{P\to V}^{2}\boldsymbol{+}\sigma_{\boldsymbol{N}}^{2} \right)$ | **(S7)** |
| --- | --- |

Therefore, if *W* is larger than the optimal solution $W_{\Delta V}=0$ (equation 7 in the main text), the variability of the associated movement vector estimation is larger than the variability of the optimal MLP solution $\sigma_{\boldsymbol{\Delta}}^{2}=\sigma_{\Delta P}^{2}$ (Supplementary Figure 1). A positive correlation between *W* and $\sigma_{\boldsymbol{\Delta}}^{2}$ is hence to be expected.

| 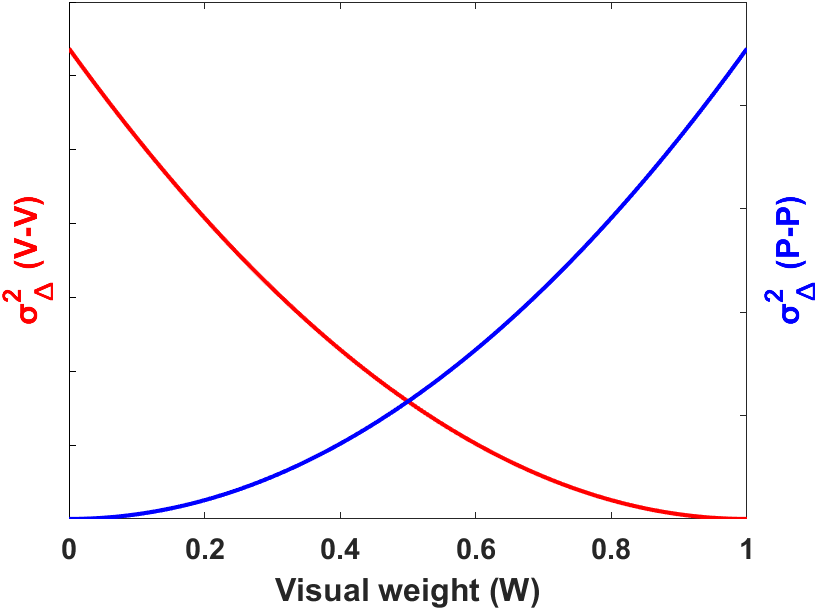 | **Supplementary Figure 1.** Graphical representation of the variance value for the unimodal V-V task (see equation S6) and P-P task (see equation S7), depending on the visual weight W.  The minimal variance is observed for the optimal weights, as described in the main text (equations 6 and 7). |
| --- | --- |

# Model predictions for the difference between seated and supine postures

Based on the optimal equations for multi-sensory integration (see equations 5-7 in the Manuscript), we present here the equations for the difference between the Supine and Seated posture predicted by the model for the movement execution variance, $D\sigma_{ME}^{2}$, and for the weight associated with visual representation of the task, $DW_{\Delta V}$, for the ‘Neck1’, ‘Neck2’ and ‘Gravity’ hypotheses.

## Unimodal tasks

For unimodal tasks (V-V and P-P), since no cross-modal sensory transformations are involved, the equations of both movement execution variance and visual weight do not contain $\sigma_{N}^{2}$ terms representing the effect of head tilt and/or neck flexions on sensory transformation.

| V-V: $\left\{ \begin{aligned} \sigma_{\Delta,Seated}^{2}=\sigma_{T_{V}}^{2}+\sigma_{H_{V}}^{2} \\ \sigma_{\Delta,Supine}^{2}=\sigma_{T_{V}}^{2}+\sigma_{H_{V}}^{2} \end{aligned} \right.$ | **(S8)** |
| --- | --- |
| P-P: $\left\{ \begin{aligned} \sigma_{\Delta,Seated}^{2}=\sigma_{T_{P}}^{2}+\sigma_{H_{P}}^{2} \\ \sigma_{\Delta,Supine}^{2}=\sigma_{T_{P}}^{2}+\sigma_{H_{P}}^{2} \end{aligned} \right.$ | **(S9)** |

It follows that all the three hypotheses predict no effect of posture on the subject performances for both unimodal tasks:

|  | $D\sigma_{ME}^{2}=0$  $DW_{\Delta V}=0$ | **(S10)** |
| --- | --- | --- |

## Cross-modal visuo-proprioceptive task

For the cross-modal task (V-P), the three hypotheses differ by where the noise ($\sigma_{N}^{2}$) added to the cross-modal sensory transformations is involved. For simplicity we assume that noise associated with the transformations from vision to proprioception of the target information ($\sigma_{V\to P}^{2})$ and from proprioception to vision of the reaching hand information $(\sigma_{P\to V}^{2})$ are characterized by a similar variance.

| $\sigma_{V\to P}^{2}=\sigma_{P\to V}^{2}=\sigma_{T}^{2}$ | **(S11)** |
| --- | --- |

### Neck1 hypothesis

According to Neck1 hypothesis, noise ($\sigma_{N}^{2}$) should be added to the cross-modal sensory transformations when the neck is laterally flexed, that is during the response phase $(\sigma_{P\to V}^{2}+\sigma_{N}^{2})$, and not during the target memorization $(\sigma_{V\to P}^{2})$, and this regardless of the body posture.

For the variances, we have:

| $\left\{ \begin{aligned} \sigma_{ME,Seated}^{2}=\sigma_{\Delta,Seated}^{2}+\sigma_{m}^{2} \\ \sigma_{ME,Supine}^{2}=\sigma_{\Delta,Supine}^{2}+\sigma_{m}^{2} \end{aligned} \right.$ | **(S12)** |
| --- | --- |

with:

| $\left\{ \begin{aligned} \sigma_{\Delta,Seated}^{2}=\sigma_{T_{V}}^{2}+\sigma_{H_{P}}^{2}+\frac{\sigma_{V\to P}^{2}(\sigma_{P\to V}^{2}+\sigma_{N}^{2})}{\sigma_{V\to P}^{2}+\sigma_{P\to V}^{2}+\sigma_{N}^{2}} \\ \sigma_{\Delta,Supine}^{2}=\sigma_{T_{V}}^{2}+\sigma_{H_{P}}^{2}+\frac{\sigma_{V\to P}^{2}(\sigma_{P\to V}^{2}+\sigma_{N}^{2})}{\sigma_{V\to P}^{2}+\sigma_{P\to V}^{2}+\sigma_{N}^{2}} \end{aligned} \right.$ | **(S13)** |
| --- | --- |

Then, $D\sigma_{ME}^{2}=\sigma_{ME,Supine}^{2}-\sigma_{ME,Seated}^{2}$ is:

|  | $D\sigma_{ME}^{2}=0$ | **(S14)** |
| --- | --- | --- |

For the visual weights, we have:

| $\left\{ \begin{aligned} W_{\Delta V,Seated}=\frac{\sigma_{V\to P}^{2}}{\sigma_{V\to P}^{2}+\sigma_{P\to V}^{2}+\sigma_{N}^{2}} \\ W_{\Delta V,Supine}=\frac{\sigma_{V\to P}^{2}}{\sigma_{V\to P}^{2}+\sigma_{P\to V}^{2}+\sigma_{N}^{2}} \end{aligned} \right.$ | **(S15)** |
| --- | --- |

Then, $DW_{\Delta V}=W_{\Delta V,Supine}-W_{\Delta V,Seated} is$:

|  | $DW_{\Delta V}=0$ | **(S16)** |
| --- | --- | --- |

The Neck1 hypothesis predicts no difference between postures in terms of variance and visual weight.

### Neck2 hypothesis

According to Neck2 hypothesis, noise ($\sigma_{N}^{2}$) should be added to the cross-modal sensory transformations when the neck muscles are active against gravity, that is when the neck is laterally flexed during the response phase of the seated condition $(\sigma_{P\to V}^{2}+\sigma_{N}^{2})$. No additional noise is added during the memorization phase of the Seated condition ($\sigma_{V\to P}^{2}$) and during both task phases in the Supine condition ($\sigma_{V\to P}^{2}$ and $\sigma_{P\to V}^{2}$).

For the variances, we have:

| $\left\{ \begin{aligned} \begin{matrix} \sigma_{\Delta,Seated}^{2}=\sigma_{T_{V}}^{2}+\sigma_{H_{P}}^{2}+\frac{\sigma_{V\to P}^{2}(\sigma_{P\to V}^{2}+\sigma_{N}^{2})}{\sigma_{V\to P}^{2}+\sigma_{P\to V}^{2}+\sigma_{N}^{2}} \\ \sigma_{\Delta,Supine}^{2}=\sigma_{T_{V}}^{2}+\sigma_{H_{P}}^{2}+\frac{\sigma_{V\to P}^{2}\sigma_{P\to V}^{2}}{\sigma_{V\to P}^{2}+\sigma_{P\to V}^{2}} \end{matrix} \end{aligned} \right.$ | **(S18)** |
| --- | --- |

Therefore, $D\sigma_{ME}^{2}=\frac{\sigma_{V\to P}^{2}\sigma_{P\to V}^{2}}{\sigma_{V\to P}^{2}+\sigma_{P\to V}^{2}}-\frac{\sigma_{V\to P}^{2}(\sigma_{P\to V}^{2}+\sigma_{N}^{2})}{\sigma_{V\to P}^{2}+\sigma_{P\to V}^{2}+\sigma_{N}^{2}}$

If we apply S11, we can simplify $D\sigma_{ME}^{2}$ as follows:

|  | $D\sigma_{ME}^{2}=-\frac{\sigma_{T}^{2}\sigma_{N}^{2}}{2(2\sigma_{T}^{2}+\sigma_{N}^{2})}$ | **(S18)** |
| --- | --- | --- |

For the visual weights, we have:

| $\left\{ \begin{aligned} \begin{matrix} W_{\Delta V,Seated}=\frac{\sigma_{V\to P}^{2}}{\sigma_{V\to P}^{2}+\sigma_{P\to V}^{2}+\sigma_{N}^{2}} \\ W_{\Delta V,Supine}=\frac{\sigma_{V\to P}^{2}}{\sigma_{V\to P}^{2}+\sigma_{P\to V}^{2}} \end{matrix} \end{aligned} \right.$ | **(S19)** |
| --- | --- |

Therefore, $DW_{\Delta V}=\frac{\sigma_{V\to P}^{2}}{\sigma_{V\to P}^{2}+\sigma_{P\to V}^{2}}-\frac{\sigma_{V\to P}^{2}}{\sigma_{V\to P}^{2}+\sigma_{P\to V}^{2}+\sigma_{N}^{2}}$

If we apply S11, we can simplify $DW_{\Delta V}$ as follows:

|  | $DW_{\Delta V}=\frac{\sigma_{N}^{2}}{2(2\sigma_{T}^{2}+\sigma_{N}^{2})}$ | **(S20)** |
| --- | --- | --- |

The Neck2 hypothesis predicts for the supine posture a smaller variance, and a larger visual weight, than in the seated position.

### Gravity hypothesis

According to the Gravity hypothesis, noise ($\sigma_{N}^{2}$) should be added to the cross-modal sensory transformations when the head is not aligned with the gravity vector, that is when the neck is laterally flexed during the response phase in the Seated condition ($\sigma_{P\to V}^{2}+\sigma_{N}^{2}$), and during both task’s phases in the Supine condition ($\sigma_{V\to P}^{2}+\sigma_{N}^{2}$ and $\sigma_{P\to V}^{2}+\sigma_{N}^{2}$). No noise is added during the target memorization phase in the Seated condition ($\sigma_{V\to P}^{2}$).

For the variances, we have:

| $\left\{ \begin{aligned} \begin{matrix} \sigma_{\Delta,Seated}^{2}=\sigma_{T_{V}}^{2}+\sigma_{H_{P}}^{2}+\frac{\sigma_{V\to P}^{2}(\sigma_{P\to V}^{2}+\sigma_{N}^{2})}{\sigma_{V\to P}^{2}+\sigma_{P\to V}^{2}+\sigma_{N}^{2}} \\ \sigma_{\Delta,Supine}^{2}=\sigma_{T_{V}}^{2}+\sigma_{H_{P}}^{2}+\frac{(\sigma_{V\to P}^{2}+\sigma_{N}^{2})(\sigma_{P\to V}^{2}+\sigma_{N}^{2})}{\sigma_{V\to P}^{2}+\sigma_{P\to V}^{2}+2\sigma_{N}^{2}} \end{matrix} \end{aligned} \right.$ | **(S21)** |
| --- | --- |

Therefore, $D\sigma_{ME}^{2}=\frac{(\sigma_{V\to P}^{2}+\sigma_{N}^{2})(\sigma_{P\to V}^{2}+\sigma_{N}^{2})}{\sigma_{V\to P}^{2}+\sigma_{P\to V}^{2}+2\sigma_{N}^{2}}-\frac{\sigma_{V\to P}^{2}(\sigma_{P\to V}^{2}+\sigma_{N}^{2})}{\sigma_{V\to P}^{2}+\sigma_{P\to V}^{2}+\sigma_{N}^{2}}$

If we apply S11, we can simplify $D\sigma_{ME}^{2}$ as follows:

|  | $\sigma_{ME}^{2}=\frac{\sigma_{N}^{2}(\sigma_{T}^{2}+\sigma_{N}^{2})}{2(2\sigma_{T}^{2}+\sigma_{N}^{2})}$ | **(S22)** |
| --- | --- | --- |

For the visual weights, we have:

| $\left\{ \begin{aligned} W_{\Delta V,Seated}=\frac{\sigma_{V\to P}^{2}}{\sigma_{V\to P}^{2}+\sigma_{P\to V}^{2}+\sigma_{N}^{2}} \\ W_{\Delta V,Supine}=\frac{\sigma_{V\to P}^{2}+\sigma_{N}^{2}}{\sigma_{V\to P}^{2}+\sigma_{P\to V}^{2}+2\sigma_{N}^{2}} \end{aligned} \right.$ | **(S23)** |
| --- | --- |

Therefore, $DW_{\Delta V}=\frac{\sigma_{V\to P}^{2}+\sigma_{N}^{2}}{\sigma_{V\to P}^{2}+\sigma_{P\to V}^{2}+2\sigma_{N}^{2}}-\frac{\sigma_{V\to P}^{2}}{\sigma_{V\to P}^{2}+\sigma_{P\to V}^{2}+\sigma_{N}^{2}}$

If we apply S11, we can simplify $DW_{\Delta V}$ as follows:

|  | $DW_{\Delta V}=\frac{\sigma_{N}^{2}}{2(2\sigma_{T}^{2}+\sigma_{N}^{2})}$ | **(S24)** |
| --- | --- | --- |

The Gravity hypothesis predicts a larger variance, and a larger visual weight, in the supine posture, in comparison to the seated posture.

# Fitting experimental data

## Algorithm

For fitting our model to the experimental data, we used Matlab® built-in “fmincon” function (R2019b, with the Optimization Toolbox) to minimize the l2-norm of the fitting errors, represented by the following cost functions, $cf1$ for the variance, and $cf2$ for the visual weigh:

| $cf1=\sum_{i=1}^{n} \left( DMSd-D\sigma_{ME}^{2} \right)^{2}$ and $cf2=\sum_{i=1}^{n} \left( D\omega_{V}-DW_{\Delta V} \right)^{2}$ | **(S25)** |
| --- | --- |

## Independent variables

In order to avoid data overfitting, the number of independent variables in the model was reduced to one ($\sigma_{N}^{2}$): the variance associated to the cross-modal sensory transformations ($\sigma_{T}^{2}$) was computed from Tagliabue & McIntyre (2011).

In Tagliabue & McIntyre (2011), the cross-modal task V-P, as well as the unimodal tasks P-P and V-V, were tested with a similar protocol in the seated posture, with the head straight. Using their results, and the equations for optimal sensory integration (see main text, equations 5-7), we have:

| $\left\{ \begin{aligned} \begin{matrix} \sigma_{\Delta, V-P}^{2}=\sigma_{T_{V}}^{2}+\sigma_{H_{P}}^{2}+\frac{\sigma_{V\to P}^{2}\sigma_{P\to V}^{2}}{\sigma_{V\to P}^{2}+\sigma_{P\to V}^{2}}=33.65 \\ \sigma_{\Delta,V-V}^{2}=\sigma_{T_{V}}^{2}+\sigma_{H_{V}}^{2}=16.0 \\ \sigma_{\Delta,P-P}^{2}=\sigma_{T_{P}}^{2}+\sigma_{H_{P}}^{2}=28.1 \end{matrix} \end{aligned} \right.$ | **(S26)** |
| --- | --- |

For simplicity, we assume:

| $\sigma_{T_{V}}^{2}=\sigma_{H_{V}}^{2}=\sigma_{V}^{2}$ and $\sigma_{T_{P}}^{2}=\sigma_{H_{P}}^{2}=\sigma_{P}^{2}$ | **(S27)** |
| --- | --- |

If we apply S11 and S27, the system of equations S26 becomes:

| $\left\{ \begin{aligned} \begin{matrix} \sigma_{V}^{2}+\sigma_{P}^{2}+\frac{\sigma_{T}^{2}}{2}=33.65 \\ 2\sigma_{V}^{2}=16.0 \\ 2\sigma_{P}^{2}=28.1 \end{matrix} \end{aligned} \right.$ | **(S28)** |
| --- | --- |

Therefore, we have:

| $\sigma_{T}^{2}=23.2$°^2^ | **(S29)** |
| --- | --- |
